# Supplementary material for: An Atypical Course of Cardiomyopathy Syndrome (CMS) in Farmed Atlantic Salmon (Salmo salar) Fed a Clinical Nutrition Diet
Source: Microorganisms. 2023 Dec 22;12(1):26. doi: 10.3390/microorganisms12010026 (PMC10820600; doi:10.3390/microorganisms12010026)

Supplementary Material

Supplementary Figure S1. Controls for in situ hybridization. A) PPIB – a general positive control probe. B) The atrium of the heart from PMCV-positive fish from an infection trial. C) The head kidney from PMCV-positive fish from an infection trial. D) DAPb – a general negative control probe. E) The atrium of the heart from PMCV-negative fish from an infection trial. F) The head kidney from PMCV-negative fish from an infection trial.

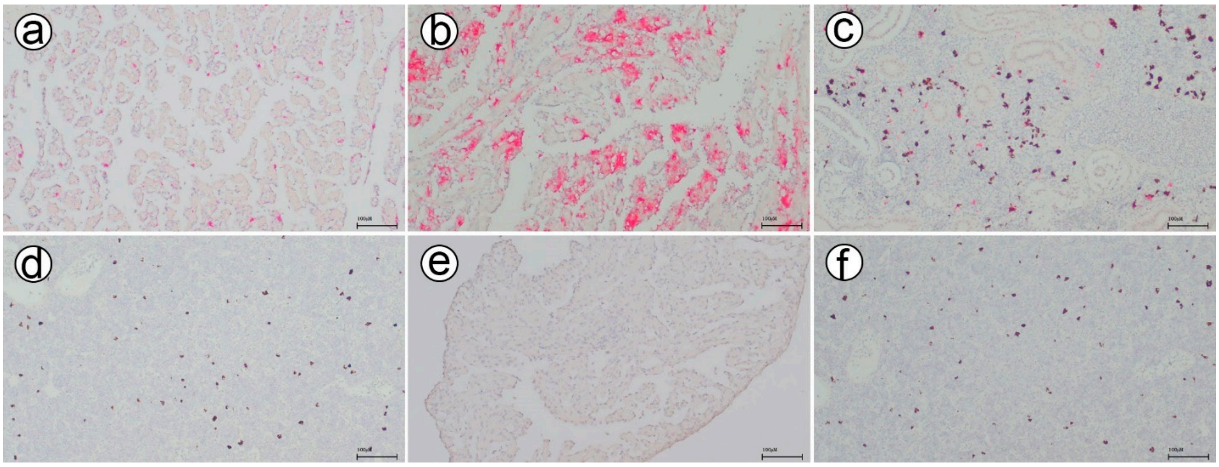

Supplementary Figure S2. Quantitative image analysis of PMCV-positive reaction (*in situ* hybridization). Red is positive labelling of PMVC, blue is the remaining tissue, and green is background of the heart sample (no tissue).

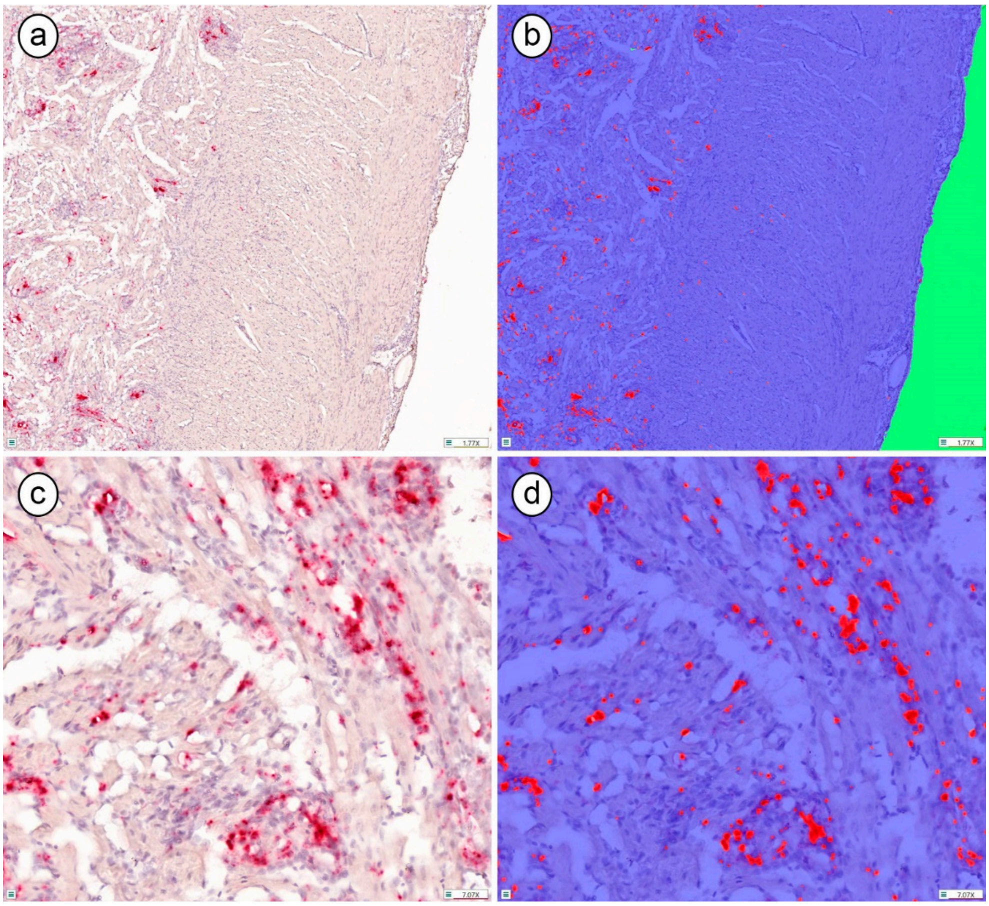

Supplementary Figure S3. Annotation of the ventricle and the atrium as different regions for quantitative image analysis.

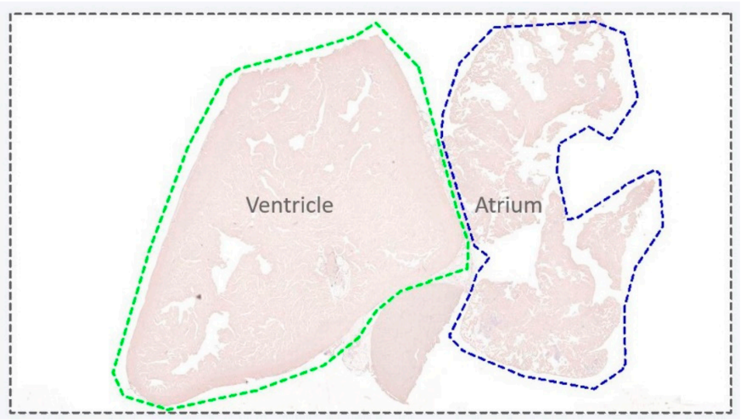

Supplement: Supplementary file 1 [file microorganisms-12-00026-s001.zip › microorganisms-2777738-supplementary.pdf]
